# Membrane-associated heparan sulfate is not required for rAAV-2 infection of human respiratory epithelia

**DOI:** 10.1186/1743-422X-3-29

**Published:** 2006-04-22

**Authors:** Michael P Boyle, Raymond A Enke, Jeffrey B Reynolds, Peter J Mogayzel, William B Guggino, Pamela L Zeitlin

**Affiliations:** 1 Department of Medicine, The Johns Hopkins University School of Medicine, Baltimore MD 21205, USA; 2 Department of Pediatrics, The Johns Hopkins University School of Medicine, Baltimore MD 21205, USA; 3 Department of Physiology, The Johns Hopkins University School of Medicine, Baltimore MD 21205, USA

## Abstract

**Background:**

Adeno-associated virus type 2 (AAV-2) attachment and internalization is thought to be mediated by host cell membrane-associated heparan sulfate proteoglycans (HSPG). Lack of HSPG on the apical membrane of respiratory epithelial cells has been identified as a reason for inefficient rAAV-2 infection in pulmonary applications in-vivo. The aim of this investigation was to determine the necessity of cell membrane HSPG for efficient infection by rAAV-2.

**Results:**

Rates of transduction with rAAV2-CMV-EGFP3 in several different immortalized airway epithelial cell lines were determined at different multiplicities of infection (MOI) before and after removal of membrane HSPG by heparinase III. Removal of HSPG decreased the efficacy of infection with rAAV2 by only 30–35% at MOI ≤ 100 for all of respiratory cell lines tested, and had even less effect at an MOI of 1000. Studies in mutant Chinese Hamster Ovary cell lines known to be completely deficient in surface HSPG also demonstrated only moderate effect of absence of HSPG on rAAV-2 infection efficacy. However, mutant CHO cells lacking all membrane proteoglycans demonstrated dramatic reduction in susceptibility to rAAV-2 infection, suggesting a role of membrane glycosaminoglycans other than HSPG in mediating rAAV-2 infection.

**Conclusion:**

Lack of cell membrane HSPG in pulmonary epithelia and other cell lines results in only moderate decrease in susceptibility to rAAV-2 infection, and this decrease may be less important at high MOIs. Other cell membrane glycosaminoglycans can play a role in permitting attachment and subsequent rAAV-2 internalization. Targeting alternative membrane glycosaminoglycans may aid in improving the efficacy of rAAV-2 for pulmonary applications.

## Introduction

Adeno-associated virus type 2 (AAV-2) is a non-enveloped parvovirus that has demonstrated efficacy as a gene replacement vector in numerous tissues [1]. As a non-enveloped virus, AAV-2 requires an extracellular receptor for attachment before internalization and intracellular processing. In 1998 Summerford and Samulski first identified heparan sulfate proteoglycans (HSPG) present on cell membrane surfaces as a receptor for AAV-2 infection [2]. Subsequent research has supported this concept, with specific heparan-binding motifs in the AAV-2 capsid recently being identified [3-5].

Cell surface heparan sulfate proteoglycans consist of complex polysaccharides linked to core proteins anchored in the cell membrane. While HSPG are characterized by repeating disaccharide units of glucosamine (GlcN) linked to glucoronic acid (GlcA) or iduronic acid (IdoA), they demonstrate dramatic variability because of differences in GlcA and IdoA content and degree of sulfation [6]. This variability allows HSPG to participate in a large number of cellular processes by binding specifically with numerous different proteins [7]. Many viruses have also been shown to bind with high affinity to HSPG, including the human immunodeficiency virus (HIV) [8], cytomegalovirus [9,10], herpes simplex virus 1 and 2 [11,12], and respiratory syncytial virus [13]. These viruses bind to cell surface HSPG for initial cellular attachment but may also utilize other cellular proteins as primary receptors for internalization. An example of this is HIV, which demonstrates high affinity binding to cell surface HSPG but uses the CD4 molecule as its primary receptor [8,14].

AAV-2 has also been demonstrated to bind with high affinity to HSPG [2,15]. The subsequent AAV-2 entry pathway is not fully understood however. After initial binding to HSPG at the cell surface, AAV-2 may engage secondary receptors which help mediate cell entry [16]. Several potential co-receptors for AAV-2 including αVβ5 integrin and human fibroblast growth receptor 1 have been suggested [17,18]. Highlighting the potential importance of other AAV-2 receptors besides HSPG is the growing number of research groups that have noted a lack of correlation for certain cell types between the quantity of HSPG present at the cell surface and susceptibility to AAV-2 infection [15]. Kern and coworkers have found that infection with rAAV-2 mutants whose capsids had been altered to dramatically reduce ability to bind to cell surface HSPG still results in remarkably high transgene expression in myocardial cells [4]. Qiu and coworkers have also noted a lack of correlation between HSPG membrane presence and rAAV-2 infection efficacy in CHO cells [15], while Opie and coworkers noted that some rAAV-2 capsid mutants unable to bind to HSPG still effectively transduce HeLa cells [3].

The cell types that have been studied for potential clinical applications of rAAV-2 gene therapy are numerous and include muscular, neuronal, retinal, hepatic and hematopoetic [1]. But rAAV-2 for gene replacement in respiratory epithelium has received particular attention because of its ability to infect cells with minimal inflammatory response [19]. While the results of some recent rAAV-2 clinical trials targeting respiratory epithelium in cystic fibrosis (CF) have been promising [20], there have also been reservations expressed about potential limitations of rAAV-2 for pulmonary use. In particular, the lack of surface HSPG on the apical membrane of respiratory epithelial cells has been identified as a serious obstacle to effective gene therapy [21,22].

The purpose of this investigation was to determine the necessity of cell membrane HSPG for efficient infection by rAAV-2, with particular attention to respiratory epithelium. The results demonstrate that while infection of cells with rAAV-2 is most efficient in the presence of membrane HSPG, absence of membrane HSPG leads to only moderately reduced infection efficiency. The results also suggest the likelihood of alternative mechanisms of rAAV-2 attachment and internalization involving other surface GAGs.

## Results

### Heparinase III treatment efficiently removes HSPG from epithelial cell membranes

**Figure 1:**
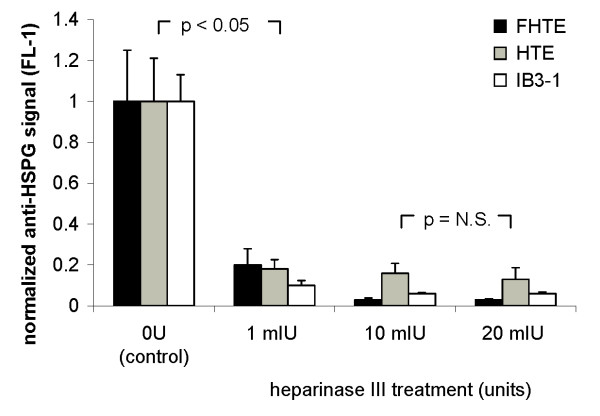
**Heparinase III treatment efficiently removes heparan sulfate from respiratory epithelial cell membranes**. Immortalized epithelial cell lines fetal human tracheal (FHTE), human tracheal (HTE), and cystic fibrosis bronchial (IB3-1) were treated with increasing amounts of heparinase III for 60 minutes, incubated with Cy3-conjugated mouse anti-heparan sulfate antibody (10E4 epitope), and assessed for HSPG surface expression by flow cytometry. Each experiment was done in triplicate and expression was normalized to mean untreated expression level. Removal of surface HSPG plateaued after treatment with 10 mIU.

To study the effects of HSPG removal on rAAV-2 infection efficacy and to ensure maximal removal of HSPG from cell membranes, human tracheal epithelial HTE cell line, fetal human tracheal epithelial FHTE cell line, and cystic fibrosis IB3-1 cell line were treated with increasing amounts of heparinase III (heparitinase) until a plateau in effect was noted. Heparinase III cleaves heparan sulfate specifically via an elimination mechanism targeting sulfated polysaccharide chains containing 1–4 linkages between hexosamines and glucuronic acid residues [2]. Cells did not demonstrate morphologic signs of cell toxicity during or after treatment. After one hour of enzyme treatment, cells were washed, then incubated with Cy3-conjugated mouse anti-heparan sulfate antibody (10E4 epitope). Membrane HSPG was then quantified by flow cytometry. This analysis demonstrated a dramatic decrease in HSPG membrane presence following treatment with even the lowest amount of heparinase III, 1 mIU (Fig. 1; amount of signal reduction in HTE 82 ± 4.6 %, FHTE 80 ± 7.9 %, IB3-1 90 ± 2.4 %, p < 0.05 for all). A ten-fold increase in the amount of heparinase III used for digestion (10 mIU) resulted in only moderate further reduction in membrane HSPG signal (HTE 84 ± 4.7 %, FHTE 97 ± 0.8 %, and IB3-1 94 ± 0.5 %). Further increases in the amount of heparinase III used for digestion (20 mIU), did not significantly further decrease the amount of membrane HSPG present (HTE 87 ± 5.7 %, FHTE 97 ± 0.4 %, IB3-1 94 ± 0.8 %, p = N.S. for all cell types) (Fig. 1). A single trial using an excessive amount of heparinase III (40 mIU) also did not demonstrate further reduction of HSPG for any of the cell lines (data not shown).

**Figure 2:**
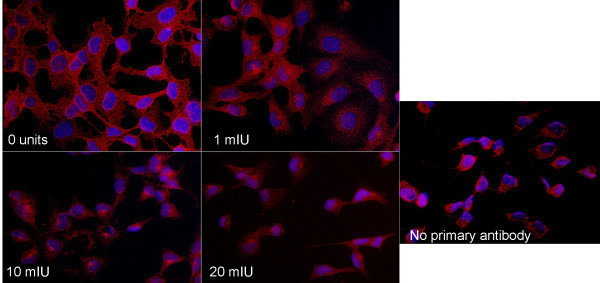
**Heparinase III treatment efficiently removes heparan sulfate from human tracheal epithelial (HTE) cell membranes**. Cells were incubated with a monoclonal mouse anti-heparan sulfate (10E4 epitope) antibody followed by a Cy3 conjugated donkey anti-mouse IgG. A DAPI nuclear counterstain was applied. Treatment of HTE cells with 10 mIU and 20 mIU of heparinase III reduced membrane HSPG staining to levels seen in cells not treated with primary anti-heparan antibody.

A reduction in cell membrane HSPG was also determined by fluorescent microscopy after treatment with monoclonal mouse anti-heparan sulfate antibody (10E4 epitope) followed by Cy3 conjugated donkey anti-mouse IgG. Consistent with flow cytometry findings, HTE cells treated with 1 mIU of heparinase III demonstrated a significant but not complete decrease in HSPG staining compared to untreated cells (Fig. 2). Treatment of HTE cells with 10 mIU and 20 mIU of heparinase III reduced membrane HSPG staining to levels seen with cells not treated with primary antibody (Fig. 2). Similar results were found in FHTE and IB3-1 cells (data not shown).

### Removal of membrane-associated HSPG from respiratory epithelial cells only moderately reduces susceptibility to r-AAV2 infection, with reduction less significant at very high MOI

**Figure 3:**
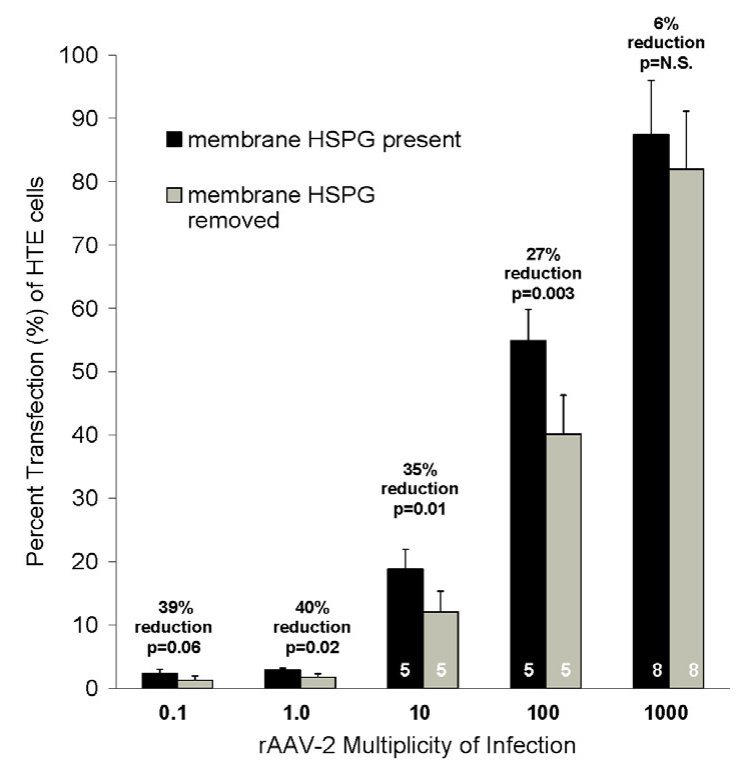
**Removal of membrane HSPG moderately reduces the percentage of human tracheal epithelial (HTE) cells transduced after rAAV-2 infection**. Treated and control HTE cells were infected with AAV2-CMV-EGFP3 for one hour after treated cells had membrane HSPG removed by a 1 hour digestion with 10 mIU of heparinase. Cells were harvested at 48 hours and immediately analyzed for percentage of cells expressing GFP by flow cytometry. Removal of HSPG reduced rAAV-2 transfection efficacy by approximately one third except at MOI of 1000. N = 3 for MOIs 0.1 and 1.0; N = 5 for MOI 10 and 100; N = 8 for MOI = 1000.

The effect of removal of surface HSPG on susceptibility to rAAV-2 infection was investigated in HTE cells by comparing transfection efficacy on heparinase treated vs control cells for a broad range of MOIs. Heparinase-treated and control HTE cells were infected with AAV2-CMV-EGFP3 at MOIs of 0, 0.1, 1, 10, 100, and 1000. At 48 hours, flow cytometry analysis demonstrated that removal of HSPG reduced rAAV-2 transfection efficacy by approximately one third for all but the highest MOI (Fig. 3; control vs heparinase treated transfection % for MOI 0.1: 2.3 ± 0.5 % vs 1.3 ± 0.5%, p = 0.06; MOI 1: 2.9 ± 0.3% vs 1.8 ± 0.5%, p = 0.02; MOI 10: 18.8 ± 3.2% vs 12.0 ± 3.3%, p = 0.01; MOI 100: 54.9 ± 4.9% vs 40.1 ± 6.2%, p = 0.003). Infections were done in triplicate for MOIs of 0.1 and 1.0, and five times for MOIs 10 and 100. At an MOI of 1000, there was less effect of removal of surface HSPG on susceptibility to rAAV-2 infection. In eight separate infections at MOI of 1000, heparinase pretreatment of HTE cells reduced AAV2-CMV-EGFP3 percent transfection by an average of only 6.1 ± 5.9% (87.4 ± 8.6% vs 82 ± 9.1%, p = N.S., Fig. 3.)

To determine if this high MOI effect was also noted in other respiratory epithelial cell lines, the same experiment was repeated at an MOI of 1000 in FHTE and IB3-1 cells. Similar results were found, with removal of membrane HSPG reducing percent transfection by only 11.8 ± 10.7% in FHTE cells and 12.7 ± 11.9% in IB3-1 cells (FHTE % transduction: 66.9 ± 14.3 % in control vs 59.9 ± 19.2% treated, n = 5 infections; IB3-1 % transduction: 51.0 ± 6.3% vs 45.5 ± 9.2%, n = 4).

### Chinese Hamster Ovary (CHO) mutant cell line pgsD-677 completely deficient in surface HSPG demonstrates only moderately decreased susceptibility to r-AAV2 infection, while CHO mutant cell line pgsA-745 deficient for all glycosaminoglycans demonstrates dramatic decrease in susceptibility

To determine if the observed moderate effect of absence of surface HSPG on rAAV-2 infection efficacy was seen in cell lines besides respiratory epithelia, we studied mutant CHO cell lines pgsD-677, previously demonstrated to be completely deficient in HSPG [23,24], and pgsA-745, deficient for *all *surface GAG. PgsD-677 is unable to produce HSPG due to a mutation which causes dysfunction of enzymes N-acetylglucosaminyltransferase and glucuronosyltransferase, both required for polymerization of heparan sulfate disaccharide chains [23,24]. PgsA-745 is deficient of all GAG due to lack of xylosyltransferase, an enzyme necessary for initiation of GAG synthesis [2,21]. The two mutant CHO cell lines were infected with AAV2-CMV-EGFP at MOIs of 10, 100, and 1000 using the techniques previously described. Transfection efficacy was again analyzed by flow cytometry and results compared to identical infection of wild type CHO-K1 cells known to have high levels of surface HSPG [23,24].

**Figure 4:**
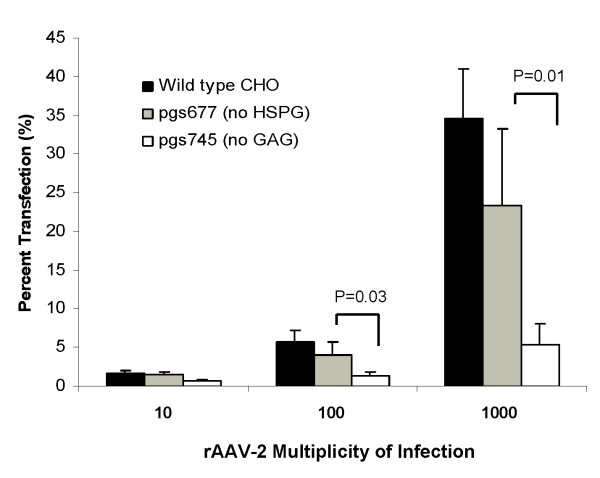
**rAAV-2 infection in CHO K1 (wild type), CHO mutant pgsD-677 (no surface HSPG) and CHO mutant pgsA-745 (deficient in all surface proteoglycans)**. CHO cell lines were infected with AAV2-CMV-EGFP at increasing MOIs. Cells were harvested at 48 hours and analyzed for percentage of cells expressing GFP by flow cytometry, with results compared between wild type CHO-K1 cells known to have high levels of surface HSPG and mutant CHO cell lines. Surface HSPG-deficient pgsD-677 transduction was reduced only 33% compared to wildtype CHO-K1 cells (pgsD % transduction at MOI 1000: 23.1 ± 6.8% vs. wildtype 34.6. ± 4.6%, p = 0.1, n = 4). In contrast, the pgsA-745 mutant cell line deficient for all surface GAG demonstrated a dramatic decrease in susceptibility to rAAV-2 infection (pgsA-745 % transduction at MOI 1000: 5.4 ± 2.7%, p = 0.0001 vs. wildtype and p = 0.01 vs. pgsD-677, n = 4).

Consistent with results from the heparinase experiments in respiratory epithelial cell lines demonstrating only moderate effect of lack of surface HSPG on AAV-2 susceptibility, surface HSPG-deficient pgsD-677 infection was reduced only 33% compared to wildtype CHO-K1 cells (Fig. 4, pgsD % transduction at MOI 1000: 23.1 ± 6.8% vs. wildtype 34.6. ± 4.6%, p = 0.1, n = 4). In contrast, the pgsA-745 mutant cell line deficient for all surface GAG demonstrated a decrease of 95% in susceptibility to rAAV-2 infection (Fig. 4, pgsA-745 % transduction at MOI 1000: 5.4 ± 2.7%, p = 0.0001 vs. wildtype and p = 0.01 vs. pgsD-677, n = 4).

### Cleavage of membrane-associated chondroitin sulfate does not alter susceptibility to rAAV-2 infection of human airway epithelial cells

In light of the observations in the total proteoglycan deficient pgsA-745 cell line, we explored the potential involvement of other GAG in rAAV-2 infection, particularly chondroitin sulfate, a glycosaminoglycan similar to heparan sulfate. Similar to the heparinase III experiments, HTE cells were treated with chondroitinase ABC prior to rAAV-2 infection. Chondroitinase ABC specifically cleaves chondroitin sulfate A, B, and C while leaving other GAGs unaltered [25]. Utilizing the previously described techniques, HTE cells were treated prior to AAV infection with either nothing (control), 6 IU of chondroitinase, 6 IU of heparinase, or both chondroitinase and heparinase. After one hour enzyme was removed and cells infected with AAV2-CMV-EGFP3 at an MOI of 100. After 48 hours cells were analyzed for GFP transgene activity by flow cytometry as previously described.

While pretreatment with heparinase alone reduced percent transduction an average of 26.6 % (40.4 ± 4.5 % transduction after heparinase treatment vs 55.1 ± 1.8 % control infection), pretreatment with chondroitinase ABC alone did not affect percent transduction at all (58.0 ± 6.0 %). Pretreatment with both heparinase and chondroitinase did not result in any further reduction in percent transduction than heparinase alone (44.9 ± 1.1 %). Similar results were seen with infections at MOI of 10 (data not shown).

## Discussion

The clinical implications of fully understanding the relationship between membrane HSPG and susceptibility to rAAV-2 infection are particularly important for pulmonary applications because of the known lack of surface HSPG on apical membranes of respiratory epithelial cells [21,22]. The purpose of this investigation was to better delineate the necessity of membrane HSPG for efficient infection by rAAV-2. Our results suggest that while infection with rAAV-2 is most efficient when membrane HSPG is present, absence of membrane HSPG only moderately reduces rAAV-2 infection efficacy. Results also suggest that the effect of absence of membrane HSPG may be smaller at high MOIs, and that other membrane glycosaminoglycans play a role in mediating rAAV-2 infection.

It is well-established that rAAV-2 binds to cell membranes utilizing HSPG as a receptor. Several investigations have confirmed specific heparan-binding motifs in the rAAV-2 capsid [3-5]. But investigators have also noted in several cell types a lack of relationship between both the amount of membrane HSPG and the ability of rAAV-2 to bind to membrane HSPG with susceptibility to rAAV-2 infection. This lack of relationship has been noted in myocardial, pulmonary, and renal cells [4]. Similar results have been noted in HeLa and CHO cells following infection by rAAV-2 with a capsid altered to be unable to bind to HSPG [3,15].

Our results are consistent with these recent observations that membrane HSPG may not be required for cell lines to be susceptible to infection and transduction by rAAV-2. Even after cell-membrane surface HSPG removal was confirmed by flow cytometry and immunohistochemistry, respiratory epithelial cell lines could be effectively transduced by rAAV-2 across a broad range of MOIs. On average, removal of cell membrane HSPG reduced rAAV-2 infection efficacy by only approximately 30–35%. This suggests that non-HSPG mediated pathways exist which permit not only cell entry, but subsequent transduction. This distinction is important because of previous observations that some cellular entry pathways may not result in nuclear entry and transduction [22].

One potential limitation to this investigation is that the rAAV-2 infections were performed on non-polarized cell populations. If HSPG-independent rAAV-2 cell entry mechanisms differ between apical and non-apical membranes, the results of this investigation may not be fully applicable for in-vivo situations. However, the infection techniques used in this study are identical to the original investigations suggesting a direct correlation between level of surface HSPG and susceptibility to rAAV-2 infection [2].

The largest effect of removal of cell membrane HSPG on rAAV-2 infection efficacy was seen at the lowest MOIs. This was in contrast to the observed small effect of removal of cell membrane HSPG at an MOI of 1000. At this MOI, infection efficacy was reduced in the three respiratory epithelial cell lines on average by only 10.2 ± 7.9 % (6.1 ± 5.9 % for HTE, 11.8 ± 10.7 % for FHTE, and 12.7 ± 11.9 % for IB3-1). These results suggest that very high MOIs may permit more non-HSPG mediated entry of rAAV-2 into cells.

One potential explanation for the observed moderate effect of removal of HSPG might be that a small amount of HSPG remained after heparinase III treatment which was not detected by immunohistochemistry or flow cytometry. To assure that the moderate effect was not due to residual surface HSPG, similar experiments were repeated in CHO cell mutants known to be completely deficient for cell membrane HSPG [24]. The results of these studies also demonstrated only a moderate effect of removal of HSPG, with AAV-2 infection again reduced on average by approximately 30% in HSPG-deficient PgsD-677 CHO cells when compared to wild-type CHO-K1 cells. The smaller effect of HSPG removal on infection efficacy at high MOI observed in respiratory epithelial cell lines was not noted in the CHO cell lines, suggesting that this high MOI effect is either unique to respiratory epithelial cell lines or was caused by a small amount of residual surface HSPG undigested by heparinase III.

The results also suggest that the non-HSPG mediated pathway involves other surface glycosaminoglycans besides HSPG. Mutant CHO cell line PgsA-745 known to be deficient for all cell membrane GAGs demonstrated a dramatic 80% reduction in susceptibility to rAAV-2 transduction. This implies that other GAGs besides HSPG can be involved in cell entry and transduction. While rAAV-2 specifically binds to HSPG, there is either another surface glycosaminoglycan that can specifically bind to rAAV-2 or GAGs that non-specifically bind to rAAV-2 and help mediate infection.

One of the proteoglycans most similar to HSPG in structure that would be a strong candidate to play a role in rAAV-2 infection is chondroitin sulfate. The HSPG-deficient CHO cell line PgsD-677 which demonstrated only moderate reduction in susceptibility to rAAV-2 infection is known to have no HSPG but produce three times as much chondroitin sulfate as wild-type CHO-K1 cells. However, removal of cell membrane chondroitin sulfate from human tracheal epithelial cells with chondroitinase ABC did not affect rAAV-2 infection efficacy at all, nor did its removal add to the effect of removal of cell membrane HSPG. This suggests that any role of chondroitin sulfate in rAAV-2 infection would be through non-specific binding to GAGs.

What are the practical implications of these results? First, lack of membrane HSPG by tissues may not preclude them from being effectively treated with rAAV-2. Using higher MOIs may permit greater utilization of non-HSPG mediated entry pathways and result in greater efficacy. Second, greater understanding of the role of other membrane GAGs in mediating rAAV-2 infection is needed. Better delineation of the non-HSPG mediated pathways available to rAAV-2 could result in new cell targeting strategies which improve efficacy of infection.

## Conclusion

Overall, our studies demonstrate that for many cell lines, lack of cell membrane HSPG results in only a moderate decrease in susceptibility to rAAV-2 infection. Other cell membrane GAGs can play a role in permitting attachment and subsequent rAAV-2 internalization. Whether this interaction involves specific or non-specific binding, or varies for apical and non-apical cell membrane locations requires further investigation. In respiratory epithelial cells, HSPG-independent cell entry mechanisms appear be more efficient at very high MOIs and this may offer a strategy to address the lack of cell membrane HSPG on apical membranes of respiratory epithelial cells.

## Methods

### Reagents

Glycosaminoglycan-specific enzymes heparinase III (heparitinase) (H8891) and chondroitinase ABC (C3667) were purchased from Sigma (St. Louis, MO). Anti-heparan sulfate (10E4 epitope) monoclonal antibodies (370258 and 370255) were obtained from Seikagaku America (Falmouth, MA).

### Cell culture

Immortalized human cell lines Human Tracheal Epithelial (HTE), Fetal Human Tracheal Epithelial (FHTE), and cystic fibrosis bronchial epithelial (IB3-1) [26] were cultured in LHC-8 basal media (Biofluids, Rockville MD) supplemented with 5% fetal bovine serum (Sigma-Aldrich, St. Louis MO), 2.5 mg/ml amphotericin (Biofluids), 80 mg/ml tobramycin (Lilly, Indianapolis IN), 0.2 mg/ml imipenem (Merck, Whitehouse Station NJ), and 100 u/ml penicillin and streptomycin (Gibco, Carlsbad CA). Normal CHO-K1 cells, HSPG-deficient CHO cells pgsD-677, and proteoglycan-deficient CHO cells pgsA-745 [23] were cultured in HAM's F12 medium supplemented with 10% fetal bovine serum, 2.5 mg/ml amphotericin, and 100 u/ml penicillin and 100 u/ml streptomycin. All cells were incubated at 37°C under 5% CO_2_.

### Enzyme treatment

Glycosaminoglycanases were stored at -20°C and reconstituted in Dulbecco's phosphate buffered saline with calcium and magnesium. Epithelial cells were treated for 1 hour at 37°C with either heparinase III (heparitinase) or chondroitinase ABC diluted in DPBS. Following incubation, cells were rinsed three times with DPBS and examined for signs of toxicity. All enzyme concentrations are described in international units (1 IU equals 600 Sigma units).

### Heparan sulfate detection

To quantify membrane heparan sulfate, cells were chilled on ice for 30 min and carefully lifted from plates with 0.04% EDTA in calcium-free, magnesium-free PBS. Cells were gently spun down and resuspended in FITC-conjugated monoclonal mouse anti-heparan sulfate (10E4 epitope) diluted 1:100 in normal media. Following a 45-minute incubation on ice, cells were analyzed by flow cytometry for FITC intensity (FL-1). Each flow cytometry experiment was done in triplicate to assure consistency of results, and normalized to negative controls. For immunohistochemistry, cells were fixed in chilled acetone for 10 min at 4°C and blocked with 5% donkey serum for 30 min at room temperature. Each specimen was incubated with a monoclonal mouse anti-heparan sulfate (10E4 epitope) antibody diluted 1:100 in PBS followed by a Cy3 conjugated donkey anti-mouse IgG diluted 1:200 (Jackson Immunoresearch, Bar Harbor ME). A DAPI nuclear stain was also applied at a concentration of 0.3 mM. Cells were qualitatively examined with an immunofluorescence microscope (Zeiss) fitted with a far-red detector, after excitation with a Krypton/Argon laser at 570 nm.

### rAAV-2 construct

AAV2-CMV-EGFP3 was provided by the University of Pennsylvania Vector Core and was constructed by cloning the enhanced green fluorescence protein reporter gene (EGFP) to pAAV2.1, an AAV-2 cis-plasmid that contains AAV2 ITR-CMV promoter-MCS-WPRE-bGH polyA-AAV2 ITR. The vector was made by transient transfection of p600 trans, pAdΔF6, and pAAV2-CMV-EGFP3 into fifty 15-cm dishes of subconfluent 293 cells. Cells were harvested 3 days after transfection. The AAV-2 vector was purified by single-step gravity-flow heparan column as previously described [27]. An infectious center assay utilizing HeLa cell line B-50 was then used to determine the infectious particle to total viral particle ratio (1:250).

### rAAV-2 infection

Twelve-well plates were seeded with 3.8 × 10^3 ^cells and grown to 75–80% confluence. Treated and control cells were then infected with AAV2-CMV-EGFP3 at MOIs of 0, 0.1, 1, 10, 100, and 1000 in serum free media at 37°C under 5% CO_2_. MOI was calculated by utilizing the previously determined infectious to total viral particle ratio of 1:250. Virus was removed after four hours and replaced with fresh media. After a 48-hour incubation, cells were treated for 15 minutes with 400 μl of enzyme-free cell dissociation buffer (Gibco, Carlsbad, CA) and lifted from plates. The suspension was gently pipetted up and down to ensure complete removal. Suspensions were immediately analyzed for the percentage of cells expressing EGFP and EGFP intensity (FL-1) by flow cytometry. Flow cytometry was performed on a FACScan utilizing the Cell Quest data analysis package (Becton Dickenson, San Jose, CA). Each flow cytometry experiment was done in triplicate or more to assure consistency of results and normalized to negative controls.

### Statistical analysis

Results are presented as mean ± standard deviation. Comparisons between groups were made using two-sided pooled-variance t-tests with p < 0.05 considered statistically significant for all analyses. Computation was performed using STATA Statistical Software, release 8.0 (College Station, TX).

## Competing interests

The author(s) declare they have no competing interests.

## Authors' contributions

MPB designed the study, analyzed the results, and drafted the manuscript. RAE and JBR helped design and perform the studies. PJM helped analyze the results. WBG and PLZ oversaw the project design and completion, provided the resources, and aided in analyzing the results. All authors read and approved the final manuscript.
